# Novel markers of myocardial recovery in patients with left ventricular assist device: A cardiopulmonary exercise test and hemodynamic analysis

**DOI:** 10.1016/j.jhlto.2025.100444

**Published:** 2025-11-21

**Authors:** Charounipha Soydara, Ilya Giverts, Catharine L. Griskowitz, Isabela Landsteiner, Kamila Drezek, Alexandra Minasian, Tara L. Logan, Janice Camuso, Alexandra E. Dempsey, Katherine Milley, Asishana A. Osho, Eriberto Michel, Erin Coglianese, Ioannis Mastoris, David A. D’Alessandro, Corrine Y. Jurgens, Gregory D. Lewis, Van-Khue Ton

**Affiliations:** a The Mass General Brigham Heart and Vascular Institute, Massachusetts General Hospital, Boston, Massachusetts; b William F. Connell School of Nursing, Boston College, Chestnut Hill, Massachusetts; c Department of Internal Medicine, Maimonides Medical Center, Brooklyn, New York; d Department of Cardiac Surgery, Massachusetts General Hospital, Boston, Massachusetts

**Keywords:** LVAD, CPET, exercise recovery delay, pressure-flow slopes, myocardial recovery

## Abstract

**Background:**

Left ventricular assist device (LVAD)-associated myocardial recovery is possible. Beyond peak O_2_ consumption (pVO_2_), parameters obtained from cardiopulmonary exercise testing (CPET), such as post exercise oxygen uptake recovery delay (VO_2_RD), ventilatory efficiency (V_E_/VCO_2_ slope), and pressure-flow slopes from invasive hemodynamics, may offer novel insights in recovery assessment.

**Methods:**

We performed a retrospective analysis of 46 LVAD recipients with CPET (2014-2024), of whom 5 had complete recovery and LVAD explant (recovered group). A subset (*n* = 23) had invasive hemodynamics during CPET. VO_2_RD thresholds were defined as the time required for VO_2_ to fall below 12.5%, 25%, and 50% of pVO_2_ (T_12.5%_, T_25%_, T_50%_). Pressure-flow slopes were derived by regressing pulmonary artery wedge pressure (PAWP) and mean pulmonary artery pressure (PAP) against cardiac output (CO) throughout exercise.

**Results:**

Compared to nonrecovered group, recovered patients had higher left ventricular ejection fraction (47% [45-48] vs 19% [16-28], *p* = 0.001), higher pVO_2_ (14.0 [13.6-20.6] vs 11.0 [9.9-12.3] ml/kg/min, *p* = 0.007), shorter VO_2_RD thresholds (T_12.5%_: 31.4 ± 11.7 vs 47.7 ± 19.1 s, *p* = 0.03; T_25%_: 46.8 ± 7.1 vs 65.3 ± 25 s, *p* = 0.0038; T_50%_: 82 ± 10.1 vs 99.6 ± 20.9 s, *p* = 0.027), lower PAWP/CO (1.1 vs 3.6 mm Hg/liter/min, *p* = 0.003) and PAP/CO (2.4 vs 4.3 mm Hg/liter/min, *p* = 0.03) slopes. V_E_/VCO_2_ slope components in all exercise phases were similar.

**Conclusions:**

VO_2_RD and pressure-flow slopes were lower in recovered patients, and could be incorporated into a prospective wean protocol assessing for complete myocardial recovery in LVAD-supported patients.

## Graphical abstract

**Figure:**
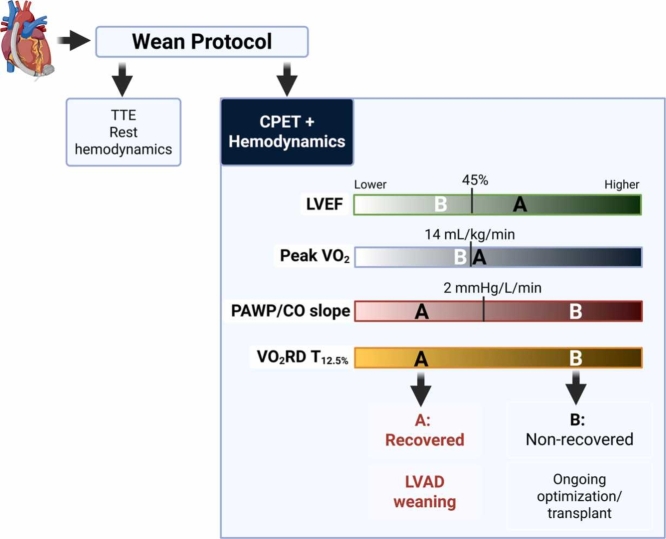
Central Illustration: Incorporation of PAWP/CO slope and VO_2_RD in a VAD Wean Protocol. Patient A and B share similar pVO_2_. Lower PAWP/CO slope and shorter VO_2_RD T_12.5%_ threshold indicate improved myocardial reserve and recovery in patient A, while elevated PAWP/CO slope and prolonged VO_2_RD indicate nonrecovery in patient B.

## Background

Durable left ventricular assist device (LVAD) promotes complete myocardial recovery in some patients with advanced heart failure (HF), allowing for successful weaning from LVAD support.^1^ Existing studies proposed echocardiographic and hemodynamic criteria at rest for LVAD weaning protocols, but few have explored the role of cardiopulmonary exercise testing (CPET) coupled with invasive hemodynamics in this context.^2^ While peak O_2_ consumption (pVO_2_) remains an important marker of global exercise capacity, little is known about the utility of other CPET parameters in evaluating for recovery in LVAD patients.

Post exercise oxygen uptake recovery delay (VO_2_RD) is defined as the time required for VO_2_ to fall permanently below peak levels after exercise cessation.^3^ In patients with HF, it is a noninvasive marker of impaired cardiac output (CO) response. Prolonged VO₂RD reflects the need to repay O_2_ deficit accumulated during exercise when CO augmentation lags behind metabolic demands, and is associated with poor hemodynamic reserve, worse HF severity, and adverse outcomes.^3^, ^4^, ^5^ VO₂RD’s role in assessing LVAD-associated myocardial recovery remains unknown.

Previous studies established that healthy controls augment intracardiac pressures in linear proportion to CO during maximal effort exercise, resulting in a pulmonary artery wedge pressure (PAWP)/CO slope ≤2 mm Hg/liter/min and mean pulmonary artery pressure (PAP)/CO slope <3 mm Hg/liter/min.^6^, ^7^ The PAWP/CO slope is consistently >2 mm Hg/liter/min in patients with HF with reduced and preserved ejection fraction (EF), and has been validated to differentiate non-LVAD HF from healthy controls in an analysis of 27 studies.^8^ A PAP/CO slope >3 mm Hg/liter/min was observed among nonrecovered LVAD patients.^9^ PAWP/CO slope ≤2 mm Hg/liter/min has previously been proposed as a criterion for LVAD explant in 2 patients at our center.^10^, ^11^ No other studies have examined whether these pressure-flow slopes could distinguish recovered from nonrecovered LVAD subjects.

Ventilatory efficiency, expressed as minute ventilation (V_E_)/carbon dioxide production (VCO_2_) slope, is related to exercise hemodynamic performance,^6^ and an elevated slope portends poor prognosis in HF patients.^12^ While V_E_/VCO_2_ slope is often measured from start to end of exercise (overall V_E_/VCO_2_ slope), prior studies demonstrated that this slope is nonlinear in certain populations.^13^, ^14^ Specifically, V_E_/VCO_2_ slope measured during submaximal exercise, that is, from the start of exercise to ventilatory anaerobic threshold (VAT) (preanaerobic threshold [pre-AT] V_E_/VCO_2_ slope), was strongly associated with exercise hemodynamic parameters and cardiovascular risk factor burden in HF with preserved EF patients and a non-HF community cohort.^13^ Whether overall and pre-AT V_E_/VCO_2_ slopes differ among recovered and nonrecovered LVAD patients have not been reported.

We hypothesized that, compared to nonrecovered patients, recovered subjects would have shorter VO₂RD, lower pressure-flow slopes, and similar V_E_/VCO_2_ slopes. These parameters may be incorporated into a wean protocol utilizing CPET and exercise hemodynamics to assess recovery.

## Methods

### Patient population

A retrospective review of our institution’s CPET database (2014-2024) yielded 46 ambulatory LVAD patients with symptom-limited CPET. Baseline characteristics, medications, New York Heart Association (NYHA) functional class, and laboratory values were collected within 2 weeks of CPET. Before 2020, patients underwent CPET only when they exhibited normalized left ventricular ejection fraction (LVEF) and were thus referred for the evaluation of recovery and potential LVAD explant. Since 2020, our institution has referred all LVAD patients to CPET once they were clinically stable and completed cardiac rehabilitation (if patients agreed and were able to exercise).

### Cardiopulmonary exercise testing

Subjects performed CPET on an upright cycle ergometer using a continuous ramp protocol (5-15 W/min) following a 3-minute unloaded pedaling phase. Exercise was continued to maximal volitional effort (respiratory exchange ratio >1.10). Breath-by-breath gas exchange data were collected using a MedGraphics metabolic cart (St. Paul, MN). We defined pVO₂ as the highest 30-second averaged VO₂ during the final minute of exercise. Oxygen uptake efficiency slope (OUES) was derived from the linear relationship between oxygen uptake (VO_2_) and the logarithm of minute ventilation (log V_E_) throughout exercise.^15^ Oxygen pulse was VO_2_/heart rate. Age-predicted maximal heart rate was 220-age (or beta-blocker–adjusted formula^16^). VAT was determined by the V-slope method.^17^ V_E_/VCO₂ slope was calculated from the start of exercise to VAT (pre-AT slope), from VAT to peak (post-AT slope), and across the entire exercise period (overall slope).^13^

### LVAD speed settings

Patients with LVEF ≥45% exercised at reduced pump speeds (HeartWare LVAD [HVAD]: 2,000 rpm, estimated flow 1.8-2.3 liter/min; HeartMate II: 8,400 rpm; HeartMate 3: 4,800 rpm, estimated flow 2.1 liter/min). Those with LVEF <45% exercised at baseline speeds. All were discharged at baseline speeds.

### Invasive hemodynamic testing

In a subset of 23 patients, invasive hemodynamic CPET in the upright position was performed as part of a wean protocol^10^ to assess for myocardial recovery. A pulmonary artery catheter was inserted via the right internal jugular vein, and a radial arterial line was placed for continuous blood pressure monitoring. Hemodynamic variables (right atrial pressure [RAP], mean PAP, PAWP) were recorded at rest and at 1-minute intervals during exercise. Fick CO was determined using measured VO₂ and arteriovenous oxygen content difference. Peripheral O_2_ extraction was determined as the difference between arterial and venous O_2_ content [C(a-v)O_2_]. RAP/CO, PAWP/CO, and PAP/CO slopes were derived by regressing RAP, PAWP, and PAP against CO over the course of exercise. Heterogeneity was corrected using the Poon’s method,^18^ where individual RAP, PAWP, PAP, and CO values were pooled for linear regression following adjustment for individual variability.

### Criteria for LVAD explant

At our institution, criteria to proceed to a wean protocol to evaluate for LVAD explant included: NYHA class I and II, mean arterial blood pressure 60 to 90 mm Hg, no uncontrolled ventricular arrhythmia, LVEF >45%, LV end-diastolic diameter (LVEDD) <60 mm, <mild mitral and tricuspid regurgitation, and normal rest hemodynamics, as previously published.^10^ Subjects fulfilling the criteria of PAWP/CO slope ≤2 mm Hg/liter/min were considered for LVAD explant.^10^, ^11^

### Derivation of VO_2_RD

VO_2_RD was defined as the time elapsed (seconds) from exercise cessation until VO_2_ permanently declined below the pVO_2_ value. To account for variation in resting VO_2_, 3 recovery thresholds were evaluated: T_12.5%_, T_25%_, and T_50%_ were defined as the time required for VO₂ to decline by 12.5%, 25%, and 50%, respectively, from pVO₂ toward each subject’s resting VO₂ level, and calculated as T_12.5, 25 or 50%_ = pVO₂ − [(0.125 or 0.25 or 0.5) × (pVO_2_-resting VO_2_)].

### Statistical analysis

Continuous variables were assessed for normality using skewness, kurtosis, and Shapiro-Wilk tests. Normally distributed variables were compared using Student’s *t*-test, and non-normally distributed variables were compared using the Wilcoxon rank-sum test. Categorical variables were compared using chi-square or Fisher’s exact tests. Effect sizes for between-group comparisons were estimated using rank-biserial correlation (for Wilcoxon tests) and Cohen’s *d* (for *t*-tests). Normality of T_12.5%_, T_25%_, and T_50%_ was assessed using histograms, Q-Q plots, and the Shapiro-Wilk test. Between-group comparisons were performed using Welch’s *t*-tests to account for unequal variances and sample sizes. Correlations between recovery thresholds and CPET or hemodynamic parameters were evaluated using Spearman’s rank correlation coefficients. All analyses were performed using Stata 17 (StataCorp, College Station, TX). Two-tailed *p* < 0.05 was statistically significant.

## Results

### Study population

Of the 46 LVAD patients included, 5 (10.9%) underwent LVAD explant due to complete myocardial recovery (recovered group), while the remainder comprised the nonrecovered group (Table 1). At time of CPET, median duration on LVAD support was 291 days, mean age was 59 ± 14 years, and 85% were male. Most had nonischemic cardiomyopathy (52.2%), NYHA class II (54.3%) symptoms, and HeartMate 3 LVAD (69.6%). Mean Charlson Comorbidity Index was 4.0 ± 1.5, indicating high comorbid illness burden. Most were on beta blockers (89%), mineralocorticoid receptor antagonists (91%), and renin-angiotensin inhibitors (RASi) (89%).

**Table 1:** Baseline Characteristics

Characteristics	Recovered (*n* = 5)	Nonrecovered (*n* = 41)	*p*-value
Age (years)	52.4±14.2	59.3±13.5	0.3
Male sex	4 (80%)	35 (85.4%)	1.0
Body mass index (kg/m²)	29.5±4.5	27.9±4.3	0.5
Type of LVAD			**0.02**
HVAD	3 (60%)	5 (12.2%)	
HeartMate II	1 (20%)	5 (12.2%)	
HeartMate 3	1 (20%)	31 (75.6%)	
Heart failure etiology			0.4
Nonischemic	4 (80%)	20 (48.8%)	
Ischemic	1 (20%)	19 (46.3%)	
Mixed	0	2 (4.9%)	
Past medical history			
Hypertension	4 (80%)	37 (90.2%)	0.5
Diabetes	1 (20%)	23 (56.1%)	0.2
Hyperlipidemia	4 (80%)	29 (70.7%)	1.0
Cardiac implantable electronic devices			**0.009**
None	4 (80%)	9 (21.9%)	
ICD	0	24 (58.5%)	
CRT-D	1 (20%	8 (19.5%)	
GDMT use at time of CPET			
Beta blocker	3 (60%)	38 (92.7%)	0.08
MRA	5 (100%)	37 (90.2%)	1.0
RASi	2 (40%)	39 (95.1%)	**0.006**
Antiarrhythmic drug	1 (20%)	19 (46.3%)	0.5
NYHA class at time of CPET			**0.03**
I	3 (60%)	4 (9.8%)	
II	1 (20%)	24 (58.5%)	
III	1 (20%)	12 (29.3%)	
IV	0	1 (2.4%)	
Echocardiographic parameters			
LVEF prior to LVAD (%)	14.4±5.9	17.4±5.8	0.3
LVEDD prior to LVAD (mm)	60±7.2	65.4±11.3	0.3
LVEF on LVAD at time of CPET (%)	47 [45-48]	19 [16-28]	**0.001**
LVEDD on LVAD at time of CPET (mm)	51.2±6.9	52.9±13.5	0.8

Abbreviations: CPET, cardiopulmonary exercise testing; CRT, cardiac resynchronization therapy; GDMT, guideline-directed medical therapy; HVAD, HeartWare LVAD; ICD, implantable cardioverter/defibrillator; LVAD, left ventricular assist device; LVEDD, left ventricular end-diastolic diameter; LVEF, left ventricular ejection fraction; MRA, mineralocorticoid receptor antagonist; NYHA, New York Heart Association; RASi, renin-angiotensin inhibitors.

Values are presented as mean ± standard deviation, median [interquartile range], or *n* (%), as appropriate.

Bold value represents p < 0.05.

Compared to nonrecovered subjects, recovered patients were more likely to have NYHA class I symptoms (60% vs 10%, *p* = 0.031), higher LVEF (47% [45-48] vs 19% [16-28] *p* = 0.001), but similar LVEDD (51.2 ± 6.9 vs 52.9 ± 13.5 mm, *p* = 0.8). No significant differences were observed in age, sex, body mass index, HF etiology, or comorbidities between the groups. Four (80%) recovered patients had no internal cardioverter/defibrillator (ICD), and 1 had cardiac resynchronization therapy (CRT-D). Among nonrecovered subjects, 24 patients (58.5%) had ICD and 8 (19.5%) had CRT-D.

### Exercise capacity

Compared to nonrecovered patients, recovered subjects had higher pVO_2_ (14.0 [13.6-20.6] vs 11.0 [9.9-12.3] ml/kg/min, *p* = 0.007), a trend toward higher OUES (1,470.1 ± 440.5 vs 1,050.5.4 ± 387.4, *p* = 0.099), less chronotropic incompetence (80.5% vs 20.0%, *p* = 0.01). Components of V_E_/VCO_2_ slope were similar: overall slope (37.2 [30.9-40.4] vs 38.8 [32.3-42.6], *p* = 0.7), pre-AT slope (29.1 [25-33.8] vs 32.3 [28-35] *p* = 0.4), post-AT slope (36.7 [35.2-37.9] vs 42.7 [36.8-54], *p* = 0.1) (Table 2).

**Table 2:** Cardiopulmonary Exercise Testing Parameters

Parameter	Recovered (*n* = 5)	Nonrecovered (*n* = 41)	*p*-value
Peak VO₂ (ml/kg/min)	14 [13.6-20.6]	11 [9.9-12.3]	**0.007**
Peak VO₂, % predicted	62 [56-80]	43 [39-51]	0.057
VO₂ recovery delay (sec)	11 [10-18]	30 [8-45]	0.2
Mean response time (sec)	91 [18-196]	103 [75-181]	0.5
Heart rate recovery at 2 minutes (bpm)	28.8±6.5	20.8±12.4	**0.049**
Peak O₂ pulse (ml/beat)	9.9±3.1	8.1±2.3	0.1
OUES	1470.1±440.5	1031.4±423.7	0.099
Pre-AT V_E_/VCO₂ slope	29.1 [25-33.8]	32.3 [28-35]	0.4
Post-AT V_E_/VCO₂ slope	36.7 [35.2-37.9]	42.7 [36.8-54]	0.1
Overall V_E_/VCO₂ slope	37.2 [30.9-40.4]	38.8 [32.3-42.6]	0.7
Ventilatory anaerobic threshold (ml/kg/min)	8 [7.5-11.3]	7.9 [6.9-10]	0.3
Exercise oscillatory ventilation	0	2 (4.9%)	1.0
Chronotropic incompetence	1 (20%)	33 (80.5%)	**0.013**

Abbreviations: VE/VCO_2_ slope, ventilatory efficiency.

Values are presented as median [25th-75th interquartile range], mean ± standard deviation, or proportions.

Bold value represents p < 0.05.

**Figure 1:**
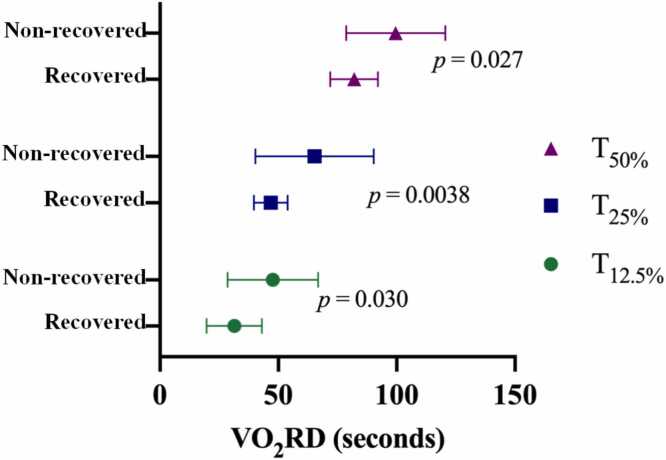
VO₂RD thresholds. Three thresholds: T_12.5%_, T_25%_, and T_50%_ are defined as the time for VO₂ to fall by 12.5%, 25%, and 50% from peak VO_2_ toward resting levels. VO_2_RD, post exercise oxygen uptake recovery delay.

Three VO_2_RD thresholds, defined by the time required for VO_2_ to decline by 12.5%, 25%, and 50% from peak toward resting VO_2_ (T_12.5%_, T_25%_, and T_50%_), were analyzed. Recovered subjects had shorter VO_2_RD across all thresholds than nonrecovered subjects (T_12.5%_: 31.4 ± 11.7 vs 47.7 ± 19.1 seconds, *p* = 0.03), T_25%_: 46.8 ± 7.1 vs 65.3 ± 25 seconds, *p* = 0.004, T_50%_: 82 ± 10.1 vs 99.6 ± 20.9 seconds, *p* = 0.03) (Figure 1).

### Pressure-flow slopes

In a subset of patients, invasive hemodynamics at rest and peak exercise in the upright position are shown in Table 3. Compared to nonrecovered group (*n* = 18), recovered subjects (*n* = 5) had similar rest and peak RAP (rest: 3 [2, 3] vs 1 [0-2] mm Hg, *p* = 0.3; peak: 12 [12] vs 12 [10.3-13.8] mm Hg, *p* = 0.7) and PAP (rest: 16 [14-18] vs 15 [13-17] mm Hg, *p* = 0.8; peak: 32 [27-38] vs 34.5 [27-39.8] mm Hg, *p* = 0.4). Rest PAWP (7 [5-7] vs 6.5 [5-8] mm Hg, *p* = 0.8) and CO (5.6 [4.3-5.7] vs 4.5 [3.7-5.2] liter/min, *p* = 0.4) were also similar. However, peak PAWP was significantly lower (16 [15-19] vs 25 [20-27] mm Hg, *p* = 0.004) and peak CO was higher (13.9 [9.9-14.6] vs 7.7 [7-10.2] liter/min, *p* = 0.04) in recovered than nonrecovered subjects.

**Table 3:** Rest and Peak Hemodynamics

Exercise phase		Recovered (*n* = 5)	Nonrecovered (*n* = 18)	*p*
Rest	RAP	3 (2-3)	1 (0-2)	0.3
	PAP	16 (14-18)	15 (13-17)	0.8
	PAWP	7 (5-7)	6.5 (5-8)	0.8
	CO	5.6 (4.3-5.7)	4.5 (3.7-5.2)	0.4
Peak	RAP	12 (12-12)	12 (10.3-13.8)	0.7
	PAP	32 (27-38)	34.5 (27-39.8)	0.4
	PAWP	16 (15-19)	25 (20-27)	**0.004**
	CO	13.9 (9.9-14.6)	7.7 (7-10.2)	**0.04**

Abbreviations: CO, cardiac output; PAP, pulmonary artery pressure; PAWP, pulmonary artery wedge pressure; RAP, right atrial pressure.

Values are presented as median (25th-75th interquartile range).

Bold value indicates significant values (*p* < 0.05).

**Figure 2:**
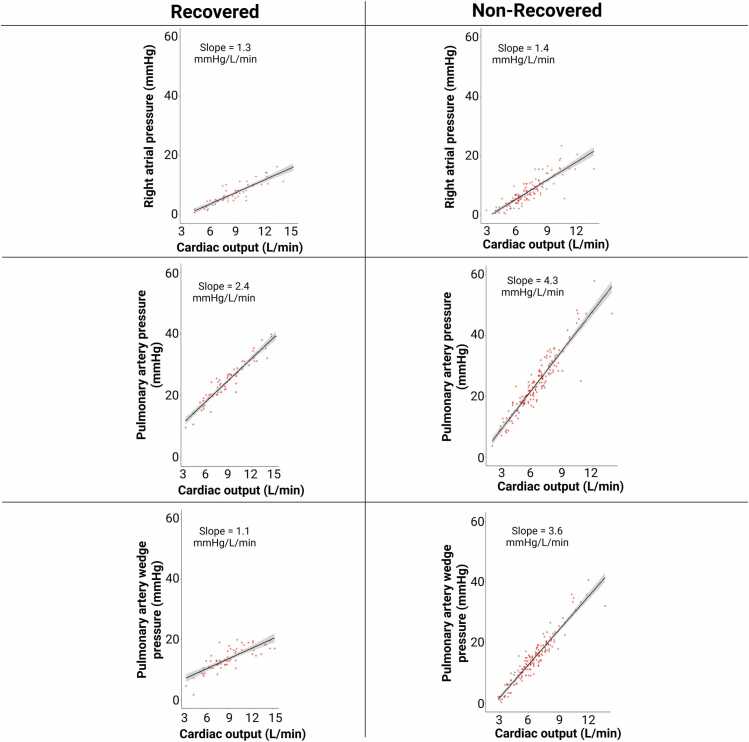
Hemodynamics throughout exercise. Poon-corrected pressure-flow slopes in recovered vs nonrecovered patients. Normal values: PAWP/CO slope ≤ 2 mm Hg/liter/min, PAP/CO slope <3 mm Hg/liter/min. CO, cardiac output; PAP, pulmonary artery pressure; PAWP, pulmonary artery wedge pressure.

Throughout exercise, RAP/CO slopes were similar between the 2 groups (1.3 vs 1.4 mm Hg/liter/min, *p* = 0.7) (Figure 2). Recovered subjects had significantly lower PAWP/CO and PAP/CO slopes than nonrecovered subjects (PAWP/CO slope: 1.1 vs 3.6 mm Hg/liter/min, *p* = 0.003; PAP/CO slope: 2.4 vs 4.3 mm Hg/liter/min, *p* = 0.03).

### Correlations between VO2RD and other CPET parameters

**Figure 3:**
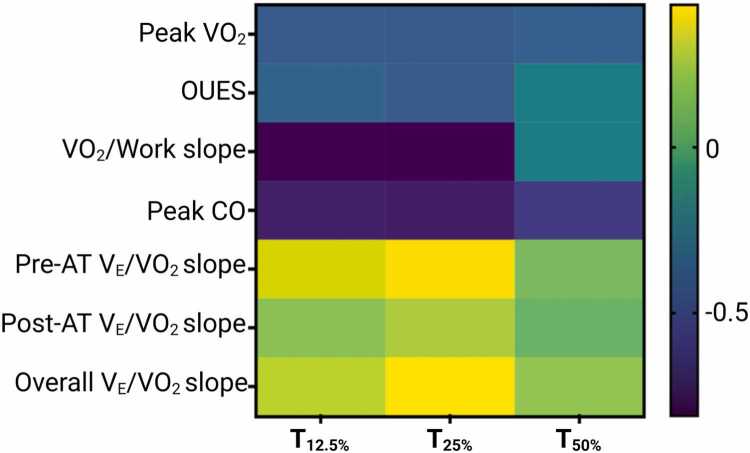
Correlation between VO_2_RD and CPET parameters. Spearman’s rank correlation coefficients between recovery thresholds (VO_2_RD) and CPET or hemodynamic parameters. Darker shades = stronger correlations. AT, anaerobic threshold; CO, cardiac output; CPET, cardiopulmonary exercise testing; OUES, oxygen uptake efficiency slope; VE/VCO_2_ slope, ventilatory efficiency; VO_2_RD, post exercise oxygen uptake recovery delay.

All 3 VO_2_RD thresholds (T_12.5%_, T_25%_, and T_50%_) inversely correlated with pVO₂ (ρ = −0.514 to −0.603), OUES (ρ = −0.433 to −0.458), and VO₂/work slope (ρ = −0.694 to −0.881). In the subset with invasive hemodynamics, VO_2_RD inversely correlated with peak CO (ρ = −0.660 to −0.778), supporting a cardiocentric mechanism for delayed VO₂ recovery in nonrecovered LVAD patients (Figure 3).

### Heterogeneity in CPET parameters

**Figure 4:**
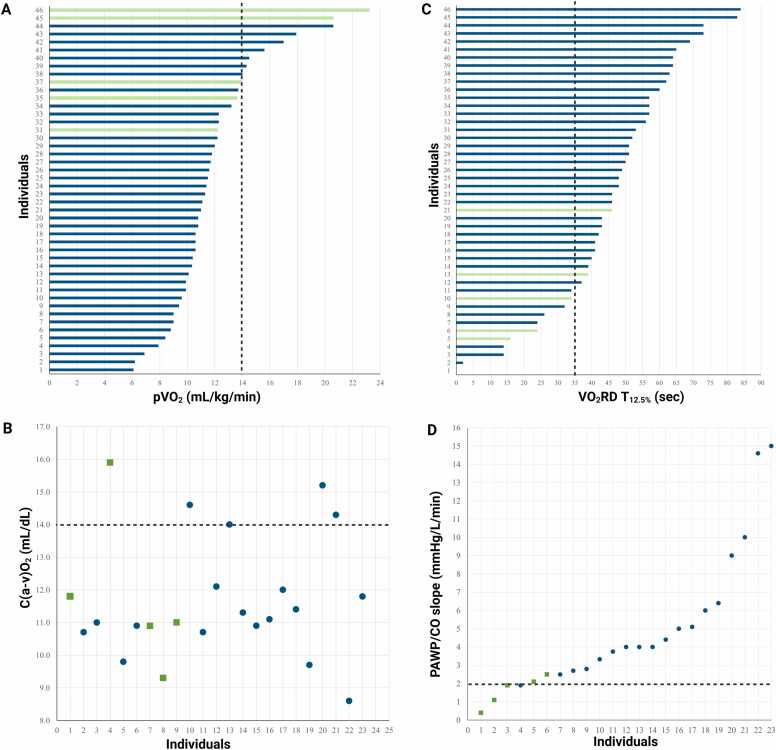
Individual CPET parameters. (A) pVO_2_ values; (B) Peripheral O_2_ extraction; (C) VO_2_RD T_12.5%_ thresholds; (D) PAWP/CO slopes. Green: recovered patients. Blue: nonrecovered patients. Dashed line: normal threshold. CO, cardiac output; CPET, cardiopulmonary exercise testing; pVO_2,_ peak O_2_ consumption_;_ VO_2_RD, post exercise oxygen uptake recovery delay.

Plots of individual pVO_2_, VO_2_RD T_12.5%_, peripheral O_2_ extraction [C(a-v)O_2_], and PAWP/CO slope revealed much heterogeneity among the cohort (Figure 4). Most subjects had persistently abnormal pVO_2_ (<14 ml/kg/min), including 3 of the 5 recovered subjects, and impaired peripheral O_2_ extraction, evidenced by C(a-v)O_2_ <14 ml/dl. In contrast, recovered subjects clustered around lower VO2RD T_12.5%_ threshold and normal PAWP/CO slopes (≤2 mm Hg/liter/min).

### Example of serial VO2RD measurements

**Figure 5:**
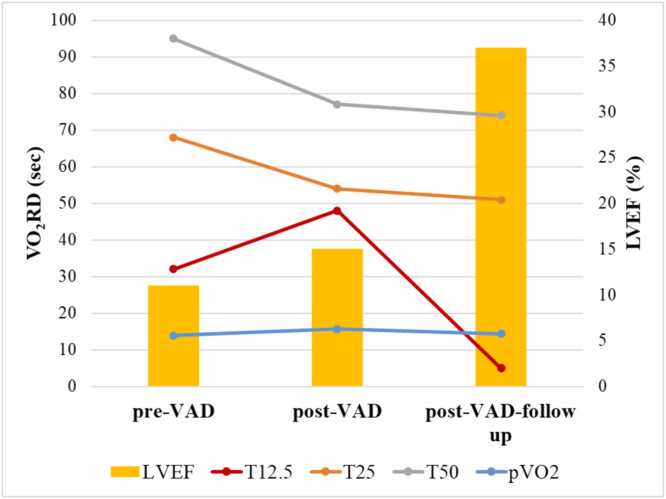
Serial VO_2_RD, LVEF, and pVO_2_ values in 1 patient. LVEF, left ventricular ejection fraction; pVO_2,_ peak O_2_ consumption; VAD, ventricular assist device; VO_2_RD, post exercise oxygen uptake recovery delay.

One of the 5 patients who underwent LVAD explant^11^ experienced HF relapse requiring LVAD reimplant 2.5 years later. Examples of serial pVO_2_, VO_2_RD, and LVEF before and after LVAD reimplant are shown in Figure 5. There was minimal change in pVO_2_ over time, while VO_2_RD appeared to track more closely with LVEF changes (shorter VO_2_RD correlating with higher LVEF).

## Discussion

We analyzed VO_2_RD, V_E_/VCO_2_ slope, and pressure-flow slopes in LVAD-supported patients with and without complete myocardial recovery. We found that compared to nonrecovered patients, recovered subjects had shorter VO₂RD, lower PAWP/CO and PAP/CO slopes, but similar RAP/CO slope and V_E_/VCO_2_ slopes. We showed that CPET parameters beyond pVO_2_ can be used to distinguish recovered from nonrecovered LVAD patients.

LVAD-induced myocardial recovery is possible.^1^, ^19^ An Interagency Registry for Mechanically Assisted Circulatory Support analysis of 13,454 patients showed that 1 in 10 patients exhibited some degree of myocardial recovery, and a select few may proceed to LVAD weaning.^1^, ^20^ Most published wean protocols relied on echocardiographic and hemodynamics at rest to determine weanability.^1^ CPET has been underutilized mainly due to insufficient data supporting specific parameter cut-offs. The only 2 CPET parameters hitherto incorporated into wean protocols were pVO_2_ and overall V_E_/VCO_2_ slope.^21^, ^22^ In the Remission for Stage D Heart Failure (RESTAGE-HF) trial, CPET was performed in all patients evaluated for LVAD weaning, but pVO_2_ > 16 ml/kg/min was not required to proceed with pump explant.^23^ LVAD patients can have low pVO_2_ due to impaired peripheral O_2_ extraction, but enough cardiac reserve to undergo successful pump explant, as shown in our study: most patients, including those with complete recovery, exhibited low C(a-v)O_2_ values.^10^ We now added to the literature by showing that, beyond the persistently low pVO_2_, VO_2_RD and pressure-flow slopes are more useful cardiocentric parameters that can be incorporated into a wean protocol utilizing CPET to differentiate recovered from nonrecovered patients (Central Illustration).

Previous works from our group and others have established that longer VO_2_RD was directly related to poor CO augmentation during exercise, and strongly predicted transplant/LVAD-free survival in patients with HF and reduced or preserved EF.^3^, ^4^, ^5^ In LVAD patients, while submaximal exercise is supported by the pump, peak exercise tolerance is primarily dependent on native CO augmentation.^9^ Therefore, it makes sense that ventilatory efficiency (pre-AT and overall V_E_/VCO_2_ slopes) was similar between our recovered and nonrecovered patients, reflecting similar submaximal exercise tolerance. In contrast, VO_2_RD would be shorter in recovered subjects, reflecting improved peak exercise tolerance and cardiac reserve.

The most widely used wean protocols relied on minimally acceptable thresholds of PAWP ≤12 to 15 mm Hg and cardiac index ≥2.4 to 2.6 liter/min/m^2^ during LVAD weaning as evidence of recovery.^23^, ^24^, ^25^, ^26^, ^27^ Our recovered and nonrecovered patients shared similar and normal PAWP and CO at rest. However, only throughout exercise did their PAWP/CO and PAP/CO slopes diverge. Recovered patients had much lower pressure-flow slopes, consistent with normalized cardiac reserve due to improved LVEF, while nonrecovered subjects had higher pressure-flow slopes, similar to those seen in non-LVAD HF patients.^6^, ^7^

Evidence supporting VO_2_RD at threshold T_12.5%_ as a noninvasive measure of cardiac reserve during exercise is accruing. A recent study from our group showed that, among 814 dyspneic patients undergoing invasive hemodynamic CPET, subjects with longer VO_2_RD at T_12.5%_ (≥35 vs <35 seconds) had higher PAWP/CO slopes.^28^ The recovered LVAD patients in this study had a mean VO_2_RD T_12.5%_ of 31.4 ± 11.7 seconds vs 47.7 ± 19.1 seconds (*p* = 0.03) in nonrecovered patients. Future studies may incorporate VO_2_RD and PAWP/CO slope in a standardized LVAD wean protocol, and explore whether LVAD therapy, coupled with specific treatments, such as intensive guideline-directed medical therapy and/or LV reloading, would shorten VO_2_RD and promote complete myocardial recovery in select subjects.

## Limitations

Data were collected retrospectively from a single center, and not all variables were available in every patient. The study population was biased toward healthier subjects who could perform symptom-limited CPET, and findings may not be generalizable in other LVAD populations. The use of a cycle ergometer for CPET may lead to an inadequate heart rate response due to reduced movement of the upper limbs in pacemaker-dependent patients, thus potentially confounding the difference in chronotropic incompetence between the 2 groups. Our cohort of 46 was either larger or on par with prior studies involving exercising LVAD patients,^9^, ^29^, ^30^, ^31^, ^32^, ^33^ but the sample size was small, and invasive hemodynamics during exercise were only available in 23 subjects. While a PAWP/CO slope ≤2 mm Hg/liter/min was previously proposed as a pump explant criterion at our center,^10^, ^11^ it did not predict HF relapse and subsequent need for LVAD reimplant in 1 patient. Additionally, the decision to explant LVAD was biased toward selecting patients with low PAWP/CO slope as evidence of adequate cardiac reserve, as demonstrated in our previously published protocol.^10^ The different use in RASi between recovered and nonrecovered subjects was likely due to the discretion of the treating physicians, and the small number of recovered patients. Unlike noninvasive CPET that can be safely and widely performed in LVAD subjects, invasive hemodynamic CPET may not be available in all LVAD centers, limiting the broad applicability of pressure-flow slopes.

## Conclusion

Our findings suggested that VO₂ recovery kinetics and pressure-flow slopes may capture impairments in cardiovascular reserve not otherwise evident in resting or peak exercise metrics. These cardiocentric parameters may be incorporated into a CPET-based wean protocol to assess for LVAD-induced myocardial recovery.

## CRediT authorship contribution statement

C.S. and V.K.T. conceived the project. C.S., I.G., C.L.G., I.L., K.D., and A.M. collected and analyzed the data. C.S. and V.K.T. wrote the manuscript. V.K.T. served as the supervising and senior author for the project. C.Y.J. and G.D.L. provided manuscript revisions. All the other authors contributed patients to the project, read and approved the manuscript.

## Declaration of Competing Interest

Dr Lewis received research funding from the 10.13039/100000002National Institutes of Health (R01-HL151841, R01-HL131029, and R01-HL159514), 10.13039/100000968American Heart Association (15GPSGC-24800006), Amgen, Cytokinetics, Applied Therapeutics, AstraZeneca, and SoniVie, honoraria for advisory boards from Pfizer, Merck, Boehringer Ingelheim, Novartis, American Regent, Cyclerion, Cytokinetics, and Amgen, and royalties from UpToDate for scientific content authorship related to exercise physiology. Dr D’Alessandro has disclosures with Abiomed (speaker's bureau) and Paragonix (equity). All the other authors have no conflicts of interest to disclose related to the contents of this manuscript.
